# Social Cognition in Schizophrenic Patients: The Effect of Semantic Content and Emotional Prosody in the Comprehension of Emotional Discourse

**DOI:** 10.3389/fpsyt.2014.00120

**Published:** 2014-09-10

**Authors:** Perrine Brazo, Virginie Beaucousin, Laurent Lecardeur, Annick Razafimandimby, Sonia Dollfus

**Affiliations:** ^1^Service de Psychiatrie, Centre Hospitalier Universitaire de Caen, Caen, France; ^2^UMR6301 Imagerie et Stratégies Thérapeutiques des Pathologies Cérébrales et Tumorales (ISTCT), ISTS Team, Université de Caen Basse-Normandie, Caen, France; ^3^Laboratoire de Psychopathologie et Neuropsychologie, Université de Paris 8, Saint Denis, France

**Keywords:** schizophrenia, social cognition, emotional discourse, semantic comprehension, emotional prosody, emotion recognition, language system

## Abstract

**Background:** The recognition of the emotion expressed during conversation relies on the integration of both semantic processing and decoding of emotional prosody. The integration of both types of elements is necessary for social interaction. No study has investigated how these processes are impaired in patients with schizophrenia during the comprehension of an emotional speech. Since patients with schizophrenia have difficulty in daily interactions, it would be of great interest to investigate how these processes are impaired. We tested the hypothesis that patients present lesser performances regarding both semantic and emotional prosodic processes during emotional speech comprehension compared with healthy participants.

**Methods:** The paradigm is based on sentences built with emotional (anger, happiness, or sadness) semantic content uttered with or without congruent emotional prosody. The study participants had to decide with which of the emotional categories each sentence corresponded.

**Results:** Patients performed significantly worse than their matched controls, even in the presence of emotional prosody, showing that their ability to understand emotional semantic content was impaired. Although prosody improved performances in both groups, it benefited the patients more than the controls.

**Conclusion:** Patients exhibited both impaired semantic and emotional prosodic comprehensions. However, they took greater advantage of emotional prosody adjunction than healthy participants. Consequently, focusing on emotional prosody during carrying may improve social communication.

## Introduction

Linguistic and neurocognitive studies have provided evidence that the recognition of emotions expressed during conversation constitutes one of the main elements of social interaction (1–3). Emotional speech comprehension in healthy participants becomes possible owing to the processing of ortholinguistic and paralinguistic information (4). Ortholinguistic processing involves the semantic language system, which represents the conceptual content (the meaning) of words, sentences, and thoughts. Elements of paralinguistic information linked to speech include gestures, facial expressions, and emotional prosody – the emotional melody of language. Emotional prosody is of special interest because it conveys information about the speaker’s emotional state, which may be relatively independent of conscious control.

Patients with schizophrenia have difficulties in daily interactions, in particular, their comprehension of speech is impaired. Most published experiments have studied the paralinguistic processes, focusing on the decoding of emotional prosodic cues. A growing body of research suggests that emotional prosodic comprehension is impaired in all patients from the early years of the illness (5–9). However, despite the impaired explicit recognition of emotional prosody, implicit processing may be preserved (10). Furthermore, patients may be more impaired when emotional prosody is ambiguous in the conversational context (11), suggesting that difficulties may lie at the level of integration of semantic and prosodic cues, rather than at the level of emotional prosodic comprehension *per se*. Previous studies have investigated the comprehension of emotional prosody in association with neutral semantic content (5–9). Disturbance of neutral semantic comprehension has been widely demonstrated in schizophrenic patients (12–16) and may be considered a trait of the illness for two reasons. First, this impairment may be associated with familial vulnerability to schizophrenia (13). Second, neither treatment of schizophrenia nor global intelligence appears to improve or worsen this deficit (14, 16). Regarding emotional utterance, only a few papers took into account the semantic comprehension of emotional content in the context of schizophrenic patients (10, 17–19). To the best of our knowledge, no studies have investigated how ortholinguistic and paralinguistic information each contribute to the comprehension of emotional discourse by schizophrenic patients.

Therefore, we aimed to assess the comprehension of emotional semantic content (ortholinguistic level), as well as the effect of additional emotional prosodic content (paralinguistic level) on comprehension, in patients with schizophrenia. The participants had to recognize the emotional content of emotional sentences including or devoid of emotional prosody. We tested the hypothesis that patients perform worse than healthy participants regarding the semantic processing of emotional sentences enunciated without emotional prosody. We also investigated whether patients were better able to classify sentences when they were enunciated with accurate emotional prosody than when they were said without emotional prosody.

## Materials and Methods

### Participants

Patients from 18 to 55 years of age were recruited from the University Hospital of Caen. They were stabilized outpatients with no change to their treatment during the last 4 months. The diagnosis of schizophrenia was established with the Mini-International Neuropsychiatric Interview (20) for the Diagnostic and Statistical Manual of Mental Disorders (21). Patients were matched to healthy volunteers on a subject-by-subject basis with regard to gender, age, and level of education. These control participants did not meet criteria for psychotic disorders or substance dependence (including alcohol) as assessed by the structured clinical interview for the DSM-IV. All participants reported French as their mother tongue and were free of neurological disorders. They granted written informed consent. The investigation was carried out in accordance with the latest version of the Declaration of Helsinski and the local ethical committee (CCP of Basse-Normandie, France) approved the study.

### Clinical and cognitive assessments

Clinical state was evaluated with the Positive and Negative Syndrome Scale [PANSS (22)]. The verbal subscale score of the Wechsler Adult Intelligence Scale-III (23) was used to evaluate global verbal skills, because intelligence quotient (IQ) is the best predictor of performance when schizophrenia patients perform emotional prosody identification tasks (24). The study participants were submitted to a detection paradigm to assess whether a general slowing of processing speed could be identified in individuals with schizophrenia. They had to detect a black cross in the middle of a white screen. Reaction time was measured using a laptop equipped with E-Prime^®^ software [version 1.2, Psychology Software Tolls, http://www.pstnet.com (25)].

### Procedure for the emotional recognition task

All sentences included words related semantically to the category to which they belonged (see Appendix for examples), and were selected to convey unambiguously emotional semantic content (26). The expressed emotions were the primary emotions such as anger, happiness, and sadness (5). The sentences were built so that the length (10 ± 2 words), word frequency, and imageability were matched across conditions. The words used were composed of two to three syllables and were frequent (2,000 occurrences in the text sample of the following database) and easily imageable (scored 5 out of 6) as regards the Brulex database criteria (26). Most of the sentences had a simple syntactic structure (subject, verb, and a complement), 5% of the sentences of each emotional category had an additional complement [see Ref. (4) for additional details about the verbal material]. Sentences were read aloud by two actors with or without emotional prosody corresponding to the emotional semantic content. This variation allows study of the facilitation effect of emotional prosody. To avoid a possible effect of the speaker’s biological sex, one half of all sentences were read aloud by an actress and other half were read by an actor (8, 27).

**Figure 1:**
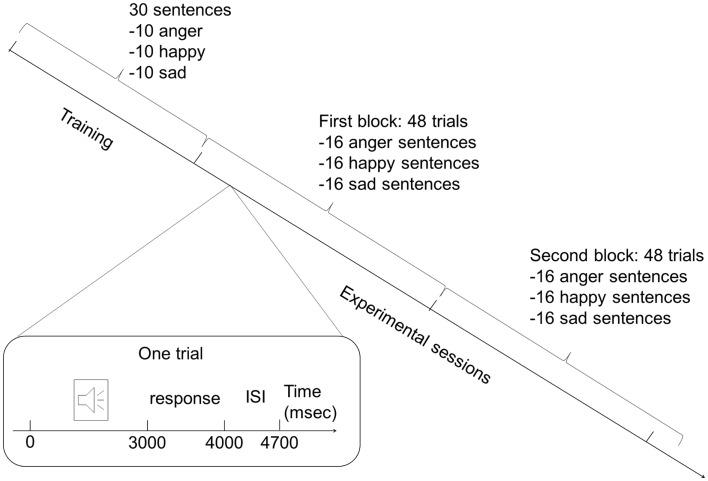
**Protocol design**. The experiment consisted of a training session and a separate experimental session. During each session, two blocks of sentences were presented: one contained sentences with emotional prosody and the other emotional prosody (order of presentation was randomly assigned to the participants). Each block during the training session contained 15 sentences (5 of each emotion), and each block during the experimental session was composed of 48 sentences (16 of each emotion). Each trial began with sentences that lasted approximately 3 s, followed by a 1-s pause for the response. Each trial ended with an interstimulus interval of 700 ms.

Each experimental session was preceded by a training session (Figure 1), which allowed study participants to become familiar with the procedure and the use of the keyboard, and allowed us to assess that the task instructions were well understood. Training consisted of 15 sentences with emotional prosody (randomly presented, 5 sentences per category) and 15 without (randomly presented, 5 sentences per category). The participants had to decide to which of the three emotional categories the sentences belonged to. After each sentence, participants pressed a button connected to a laptop, which recorded their answer. Despite that cognitive and motor slowing might affect patients, response time was limited to 1 s in order to prevent the participants from engaging in complex compensatory cognitive strategies that might mask spontaneous responses owing to a prosodic effect.

The experimental session was made up of two blocks of 48 sentences: one block with sentences expressed with emotional prosody, and the other without emotional prosody. The same number of sentences was employed to express each emotion with or without emotional prosody in order to exclude representation bias. Sentences were randomly presented in each block (16 sentences per emotional category per block). One-half of the participants in each group first assessed the block of sentences with emotional prosody, followed by the block of sentences lacking emotional prosody. The remaining participants in each group assessed the blocks of sentences in inverse order to minimize any effect of the order of presentation. The presentation order of the stimuli was randomized in each of the two blocks.

### Number of required participants

The number of required subjects was at least 14 in each group, patients, and controls, based on a previous study. This psycho-experimental study (28) gave the mean and SD of the number of correct answers in a group of control participants when sentences are enounced by actors (mean ± SD = 37 ± 1.4). Accordingly, the number of subjects in each group should be 14, postulating a difference between patients and controls of 1.5, and a SD 1.4 with α = 0.05 and β = 0.1 (29).

### Statistical analyses

A three-factor analysis of variance was used to investigate any effect of prosody on the response rate, which was defined as the percentage of correct answers based on the number of sentences delivered. The three factors were group (patients, controls), emotion (anger, happiness, sadness), and prosody (with and without emotional prosody). The verbal IQ score was taken into account as a covariate (ANCOVA). Significant interactions were analyzed further with Tukey’s post-tests. Verbal IQ and reaction time to the detection paradigm were compared between the groups using a paired *t*-test. Correlation analyses were conducted between the response rate and the symptomatic assessments (PANSS).

## Results

### Socio-demographic and clinical characteristics of participants

Data are summarized in Table 1. Sixteen participants were included in both groups. All patients were treated with antipsychotic drugs. Patients exhibited lower performances on the verbal IQ test than controls (*p* = 0.0002; η^2^ = 0.35). The reaction time to the detection paradigm did not differ significantly between groups (for the patient group, mean ± SD = 414 ± 172 ms confidence interval at 95% (CI) (330, 499) versus for the control group, 327 ± 60 ms, CI (297, 356), *p* > 0.07; η^2^ = 0.10).

**Table 1:** **Participants’ demographic and clinical characteristics**.

	Healthy controls	Patients with schizophrenia
Sex, *n*	9M/7F	9M/7F
Secondary education, *n*	7	7
Tertiary education, *n*	9	9
Age, years^a^	40.3 ± 9.3 (36, 45)	39.7 ± 8.6 (35, 44)
Age of illness onset, years^a^	–	23.2 ± 4.7 (21, 26)
Duration of illness, years^a^	–	13.3 ± 5.8 (10, 16)
Verbal IQ^a^	105.6 ± 11.6 (100, 111)	89.8 ± 11.5 (84, 95)
PANSS total score^a^	–	49.7 ± 7.2 (46, 53)
PANSS positive subscale^a^	–	14 ± 3.7 (12, 16)
PANSS negative subscale^a^	–	10.6 ± 3.4 (9, 12)
Chlorpromazine equivalent, mg^a^	–	265.5 ± 168.9 (183, 348)

*M, male; F, female; PANSS, Positive and Negative Syndrome Scale; IQ, intelligence quotient*.

*The 95% confidence intervals are given in brackets*.

*^a^Data are presented as mean ± SD*.

### Emotional speech comprehension

The descriptive analysis revealed that the patients’ response rate was inferior to the controls’ response rate (Table 2).

**Table 2:** **Performances of both groups^a^**.

	Response rate		NR	
	Prosody+	Prosody−	Prosody+	Prosody−
Healthy controls (CI 95%)	96 ± 4 (94, 98)	84 ± 7 (81, 87)	3 ± 4(2, 2)	10 ± 7(7, 14)
Patients with schizophrenia (CI 95%)	78 ± 16 (70, 86)	58 ± 19 (49, 67)	22 ± 17 (14, 30)	37 ± 20 (28, 47)

*Prosody+, with emotional prosody; Prosody−, without emotional prosody; NR, no response; CI 95% confidence interval*.

*^a^Data are presented as percentage ± SD*.

**Figure 2:**
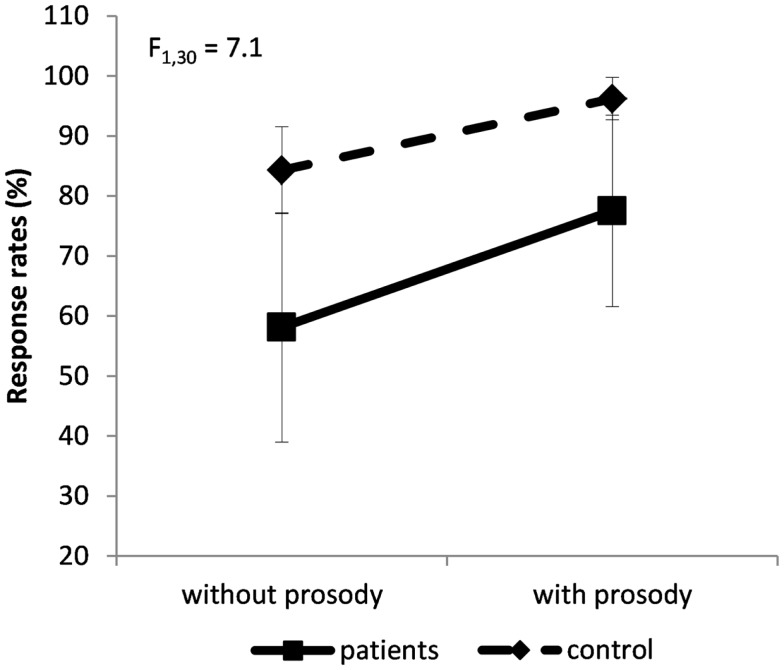
**Group × prosody interaction on the percentage of response rate (±SD)**. Dashed line for controls’ performances, solid line for patients’ performances.

The ANCOVA regarding the response rate revealed a group × prosody interaction, *F*(1,30) = 7.1; *p* = 0.01; η^2^ = 0.18 (Figure 2): schizophrenia patients performed significantly worse than controls. Even if emotional prosody facilitates the performances of both groups, it facilitated the task more in patients. For information, the main effect of group was confirmed: patients were more impaired than controls, *F*(1,28) = 12.5; *p* < 0.002; η^2^ = 0.47 (Figure 2). There was also an effect of prosody, *F*(1,30) = 118; *p* < 0.0001; η^2^ = 0.79 (Figure 2): both groups achieved higher response rates for sentences expressed with emotional prosody than for sentences expressed without emotional prosody.

**Figure 3:**
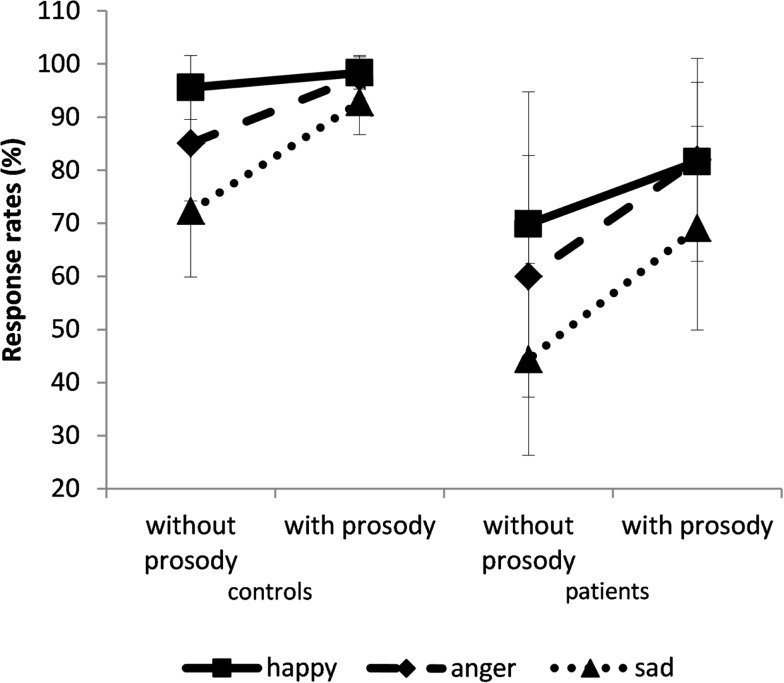
**Percentage of response rate (±SD)**. The results are given for the control (left panel) and patient groups (right panel) for sentences spoken with or without prosody. Solid line for happy, dashed line for anger, and dotted line for sadness.

There was also a prosody × emotion interaction: sadness was less recognized than other emotions, even more among the sentences that were devoid of emotional prosody, *F*(2,60) = 11.5; *p* < 0.0001; η^2^ = 0.28 (Figure 3). An emotional effect was also observed, *F*(2,60) = 47; *p* < 0.0001; η^2^ = 0.61. Happiness was better recognized than anger (*p* = 0.01), which in turn was better recognized than sadness (*p* = 0.0001). Therefore, the participants’ worst performances were achieved when classifying sad sentences.

### Correlations between task performance and symptoms

The response rate for all sentences expressed with emotional prosody (*r* = −0.5; *p* = 0.04) or without emotional prosody (*r* = −0.5; *p* = 0.05, emotional categories were collapsed) decreased with the total PANSS score. Only one correlation was observed with the positive subscale score, which correlated negatively with the classification score for angry sentences spoken with emotional prosody (*r* = −0.7; *p* = 0.006). There was no correlation with the PANSS negative subscale.

## Discussion

Patients performed significantly worse than controls even in the presence of emotional prosody, showing that their ability to understand emotional semantic content was impaired. Although prosody improved performances in both groups, it benefited the patients more than the controls. Finally, the emotional effect was observed for both patients and controls: happiness was the best-recognized emotion. Sadness was less well recognized than the other emotions, even when sentences contained emotional prosody.

### Comprehension of emotional semantic content

This study is the first to emphasize impaired emotional semantic comprehension in schizophrenia patients. Most published studies employ semantically neutral words or sentences expressed with emotional prosody (6, 9, 10, 19, 24, 30–39). Only a few protocols use stimuli with emotional semantic content (10, 17–19). However, in these studies, protocol designs did not allow researchers to focus specifically on semantic comprehension. For instance, participants were instructed to judge the emotional valence of the meaning of a word, while ignoring its emotional prosody [emotional semantic content pronounced with congruent emotional prosody versus emotional semantic content pronounced with incongruent emotional prosody; (10)]. The slowing of performance for the incongruent versus the congruent items (the vocal emotional Stroop effect) was quantified. So this experiment did not investigate participants’ comprehension of emotional semantic content, but rather their implicit recognition of emotional prosody. Therefore, unlike the present study, the study by Roux et al. (10) did not allow investigation of the comprehension of emotional semantic content expressed without any prosody. In the same vein, Huang and Mitchell’s studies included stimuli with congruent or incongruent semantic and prosodic contents that cannot fully segregate the comprehension of semantic and prosodic content (17, 18). In Scholten’s study, participants had to identify the emotion conveyed by prosody and semantic content in four types of sentences (19). This study was interesting because, to the best of our knowledge, it is the only one that included verbal material that contained emotional semantic content pronounced with neutral prosody, resembling our condition. Unfortunately, the statistical analysis was performed on collapsed performances, preventing any comparison with the present study.

### Impact of emotional prosody on emotional comprehension

The lower response rate in patients compared with controls in the congruent condition demonstrated impaired emotional prosodic comprehension among individuals with schizophrenia. These results agree with the literature regardless of the paradigm used (5–11, 17, 24, 32–34, 36, 39–42).

As our group of patients was not slowed by the detection paradigm, we conclude that their worse performances were specific to prosodic processing (10), and could not be attributed to a general slowing in processing speed in schizophrenia. The present study shows that the presence of emotional prosody improved performances in both groups, but more in patients than in controls. We raise the hypothesis that patients were able to exploit the potential interaction between emotional prosody and strong emotional semantic content more efficiently than controls. These results are of special interest because, to our knowledge, only Roux et al. (10) raised the question of the emotional semantic/prosody interaction. Using the vocal emotional Stroop task, they found that patients “made more errors overall than control participants. More errors were made on incongruent items than on congruent ones, suggesting that emotional prosody interferes with semantic judgement of emotional valence.” Our results allow us to go a step further in the interpretation. Indeed, we propose that the improvement of the patients’ response rate with emotional prosody is the expression of an efficient use of emotional prosody, which allows patients to compensate for the impairment of emotional semantic comprehension. Of course, the question of the effect of these results on everyday conversation is raised. Indeed, our paradigm focused on short, simple sentences characterized by strong congruence between semantic content and expressed emotional prosody, which did not represent the common daily social interaction.

The most pronounced prosodic impairment concerned negative emotions, particularly sadness. Although some studies have suggested that it is specific to patients (6, 30, 41), Edwards and collaborators were unable to distinguish whether the difficulty encountered by patients compared with healthy controls regarding sad stimuli was specific to this emotional category, rather than a difficulty linked to their stimuli (6). Further investigation must tackle the question of specific impairments of negative emotion recognition in patients.

### Potential covariates

While studies suggested that gender did not influence prosodic competence in schizophrenia patients (7, 9, 11) or healthy participants (43) others have shown that males (healthy participants and patients studied together as a group) were more impaired than their female counterparts (19, 42). However, in these latter studies, both male and female patients performed worse than their gender-matched control groups. Our patient and control groups were strictly matched regarding gender to avoid any kind of gender-based influence on the results.

According to some authors, global cognitive status contributed to the prediction of emotion recognition (6, 7). However, the deficit remained significant when a difference of education level and/or IQ between patients and controls was taken into account as a covariate (9, 10, 19, 33). Thus, we matched patients to healthy volunteers on a subject-by-subject basis regarding level of education and took into account verbal IQ as a covariate in our analysis, which allowed us to conclude that the impairments observed in our patients were not entirely due to general cognitive decline.

Performance may be linked to the nature (11, 19, 31, 37, 38) or severity of symptoms (10). However, in a review by Edwards et al. (5), the results with regard to the paranoid/non-paranoid distinction were mixed, and some papers sustained that there is no support for a relationship between prosody performances and symptoms (9, 36). In our study, correlation analyses showed that only global symptom severity might have a negative impact on patients’ ability to classify the emotional content of sentences; however, it was independent of prosodic status. Moreover, the weakness of the correlation suggests that the real impact of symptom severity may be minor.

### Implications of the study

The small size of our sample implies that findings must be replicated in an independent experiment. However, we stress the fact that this sample size was based on previous results, which allowed us to calculate the number of required participants in order to find if the group of patients differs from controls. So they deserve interest since, for the first time, a study shows that patients exhibited impairment regarding comprehension of the emotional semantic content of spoken sentences and that they took greater advantage of emotional prosody adjunction than healthy participants. Consequently, new therapeutic perspectives may be raised in cognitive remediation. Focusing on emotional prosody may improve social communication with schizophrenia patients.

## Conflict of Interest Statement

The authors declare that the research was conducted in the absence of any commercial or financial relationships that could be construed as a potential conflict of interest.

## Appendix

### Examples of sentences

Happy sentence: I am happy to come dining with you.

Sad sentence: I heard a very bad news, this morning.

Angry sentence: Greedy pig, he ate my favorite cakes!
